# Vitamin D receptor absence does not enhance intestinal tumorigenesis in *Apc^Pirc/+^*rats

**DOI:** 10.1242/bio.059290

**Published:** 2022-07-06

**Authors:** Amy A. Irving, Bayley J. Waters, Jeremy R. Seeman, Lori A. Plum, Hector F. DeLuca

**Affiliations:** 1 Department of Biochemistry, University of Wisconsin-Madison, 433 Babcock Drive, Madison, WI 53706, USA; 2 Organic Lab, DiaSorin Inc, 1951 Northwestern Avenue, Stillwater, MN 55082, USA

**Keywords:** Colon cancer, Vitamin D receptor, Calcium, Rat model

## Abstract

Epidemiological observations have prompted some to posit that elevated circulating vitamin D is responsible for reduced colon cancer in individuals residing near the equator. We have previously demonstrated that vitamin D has no effect on colon cancer in two rodent models of intestinal tumorigenesis. We have now extended this line of inquiry to ask whether ablation of vitamin D receptor (VDR) affects tumorigenesis. A VDR null rat was developed using Cas9-CRISPR technology, which allowed us to investigate whether 1,25(OH)D_3_ signaling through its receptor plays a role in intestinal tumorigenesis. Loss of VDR expression alone did not induce tumorigenesis, even in animals exposed to the inflammatory agent dextran sodium sulfate. These *VDR^−/−^* rats were then crossed with *Apc^Pirc/+^* rats, which are predisposed to the development of intestinal neoplasms. In combination with the *Pirc/+* mutation, VDR loss did not enhance tumor multiplicity, growth, or progression in the colon or small intestine. This study demonstrates that the vitamin D receptor does not impact tumor development, and strongly supports previous findings that vitamin D itself does not play a role in colon cancer development or progression. Alternative explanations are needed for the original latitude hypothesis, as well as observational data in humans.

This article has an associated First Person interview with the first author of the paper.

## Abstract (precis)

**Summary:** Loss of vitamin D receptor, alone or in combination with either an inflammatory agent or the *Apc^Pirc/+^* rat intestinal tumor model, did not enhance tumor occurrence, growth, or progression.

## INTRODUCTION

Over four decades ago, epidemiological observations suggested a positive correlation between colon cancer mortality and distance from the equator. Owing to the induction of vitamin D synthesis in skin by ultraviolet light exposure, a protective role for vitamin D was postulated (Garland and Garland, 1980). In support of this hypothesis, numerous studies have indeed shown that colon cancer patients tend to have lower serum vitamin D levels than do healthy controls.

In parallel to these findings, vitamin D receptor (VDR) has been proposed to play a role in colon cancer susceptibility and patient outcome. Expression of VDR actually increases in early stage hyperplastic polyps, and steadily declines throughout the later stages of tumorigenesis, with virtual absence in metastases (Sun, 2017). Additionally, some VDR polymorphisms are associated with increased risk of developing cancer (Slattery et al., 2007, 2009; Beckett et al., 2016; Cho et al., 2018). Further, VDR expression has also been inversely associated with survival (Zhu et al., 2017).

While associations between vitamin D or VDR and cancer have been shown in multiple studies, few studies exist that rigorously test whether lack of vitamin D or its receptor directly affects intestinal cancer. We have previously demonstrated in both rat and mouse models of intestinal tumorigenesis that vitamin D supplementation does not protect against intestinal tumor development; it instead enhances tumorigenesis in the colon of the rat (Irving et al., 2011, 2015). We subsequently investigated whether vitamin D deficiency increased susceptibility to tumorigenesis using several different rat models, but found no evidence to support a role for vitamin D (Irving et al., 2018). Jointly, these studies strongly suggest that vitamin D ligand does not play a direct role in intestinal tumorigenesis.

Now we have investigated a role for vitamin D from an alternate angle, by interrupting signaling of 1,25(OH)D_3_, the active metabolite, through its receptor, VDR. Development of a VDR null rat has allowed us to investigate whether 1,25(OH)D_3_ signaling through its receptor plays a role in intestinal tumorigenesis, as well as examine possible ligand-independent actions of VDR alone, which has been demonstrated for skin (Bikle, 2021). Combined with a model that promotes intestinal inflammation, the potential role of VDR as a tumor initiator was examined. By contrast, when tumors are initiated by mutation in the Adenomatous polyposis coli gene, which results in nuclear accumulation of β-catenin, we determined whether loss of vitamin D hormone signaling through VDR further promotes the growth or progression of intestinal tumors. Here, we report our extensive study of VDR in the rat. Based on the culmination of our previous work we hypothesize that loss of VDR signaling will not affect neoplastic phenotype.

## RESULTS

### Phenotyping of the VDR null rat

**Fig. 1.**
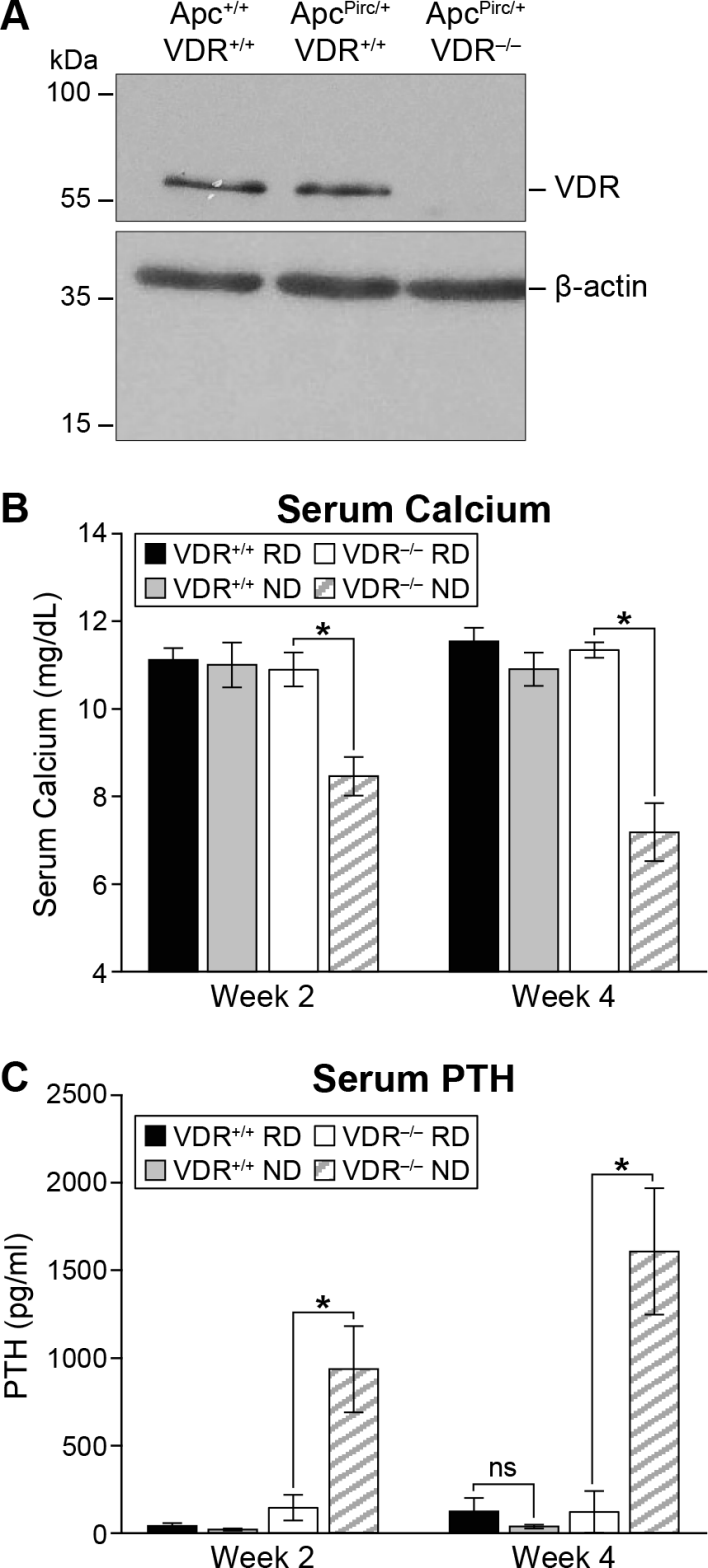
**Phenotyping of the VDR null rat.** (A) No VDR protein was detected by western blot in the colon of *VDR^−/−^* rats. (B) On an ND, *VDR^−/−^* rats show a significant reduction in serum calcium within 2 weeks, which is further reduced by 4 weeks on diet (*P*<0.0001). Feeding knockout rats RD maintains their serum calcium at normal levels comparable to wild-type animals on either diet. (C) As expected, serum PTH was significantly elevated in *VDR^−/−^* rats maintained on ND (*P*<0.001); this increase was generally abrogated when VDR^−/−^ rats were placed on RD. Black bars, *VDR^+/+^* on RD (*n*=6); gray bars, *VDR^+/+^* on ND (*n*=6); white bars, *VDR^−/−^* on RD (*n*=7); hatched bars, *VDR^−/−^* on ND (*n*=7). Serum biochemical assay results were analyzed using a two-sided Wilcoxon rank sum test. * *P*≤0.05, ns denotes no statistically significant difference. Data shown as mean±s.d.

In the VDR null rat, Sanger sequencing determined that VDR is transcribed normally until Val20, with an addition of 20 amino acids of nonsense peptide before a stop codon is encountered. This occurs before the beginning of the first annotated protein domain, a zinc finger domain. Wild-type sequence: (ATGGAGGCAACAGCGGCCAGCACCTCCCTGCCCGACCCTGGTGACTTTGACCGGAACGTGCCCCGGATCTGTGGAGTGTGTGGAGACCGAGCCACAGGCTTCCACTTCAATGCTATGACCTGTGA); mutant sequence: (ATGGAGGCAACAGCGGCCAGCACCTCCCTGCCCGACCCTGGTGACTTTGACCGGAACGT**GG**ATCTGTGGAGTGTGTGGAGACCGAGCCACAGGCTTCCACTTCAATGCTATGACCTGTGA). Western blot has confirmed a lack of VDR protein in null animals (Fig. 1A).

Rats lacking expression of VDR develop a physical phenotype very similar to what is seen in VDR null mice, with progressive loss of fur generally beginning shortly after weaning (Bikle et al., 2006). VDR null mice require maintenance on a rescue diet (RD) to maintain mineral balance (Li et al., 1998); this has also proven true in the rat. When VDR null animals are placed on a normal diet (ND), significant phenotypic effects are seen, including hypocalcemia and hyperparathyroidism.

VDR null rats maintained on RD show serum calcium and parathyroid hormone (PTH) concentrations similar to those of wild-type. However, within 2 weeks of being placed on ND, serum calcium in VDR null rats falls to 8.5±0.4 mg dl^−1^, compared to those on RD at 10.9±0.4 mg dl^−1^ (*P*<0.001, Fig. 1B). After 4 weeks on ND, serum calcium in VDR null rats falls further to 7.2±0.7 mg dl^−1^ (versus 11.3±0.2 mg dl^−1^ on RD, *P*<0.001). Simultaneously, PTH levels in VDR null rats on ND rise dramatically to 936±246 mg mg dl^−1^ at 2 weeks and 1608±361 mg dl^−1^ at 4 weeks post diet, compared to null rats on RD [146±73 mg dl^−1^ at 2 weeks (*P*=0.0005), 121±119 mg dl^−1^ at 4 weeks (*P*=0.0006), Fig. 1C]. In wild-type rats neither circulating calcium (11.0±0.5 mg dl^−1^ on ND, 11.1±0.3 mg dl^−1^ on RD, *P*=0.3) nor PTH level (20±6 mg dl^−1^ on ND, 42±15 mg dl^−1^ on RD, *P*=0.2) is affected by diet. Taken together, these results support a lack of functional VDR in the null rat.

### Tumor multiplicity and sizing data

Loss of functional VDR alone did not cause the development of tumors in either the small intestine or colon in any of 36 animals examined (six rats per sex at each 120, 150, and 180 days of age).

**Fig. 2.**
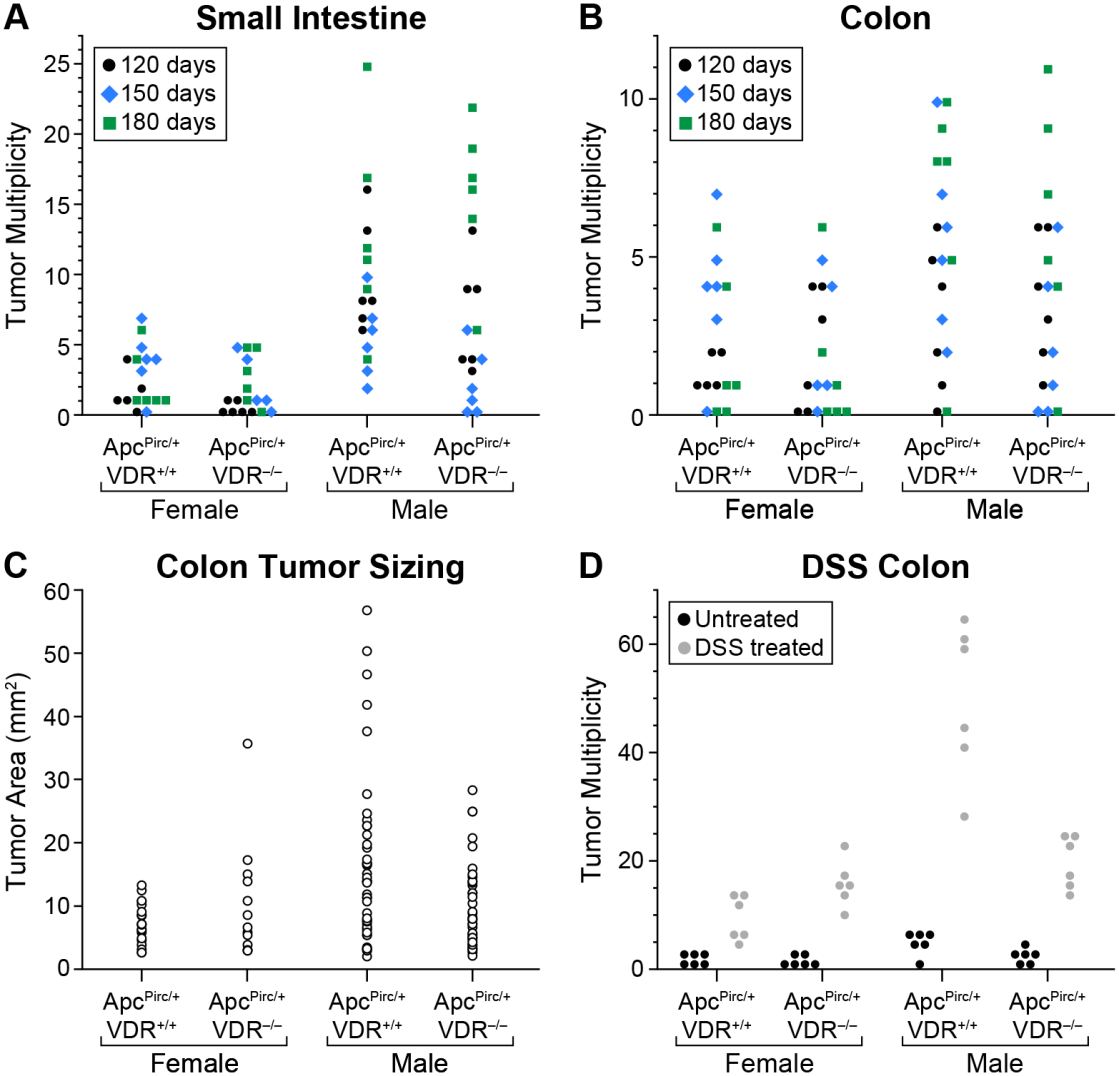
**Tumor multiplicity and size data.** *VDR^−/−^* rats with wild-type *Apc* did not develop tumors in either the small intestine or colon. (A) In the small intestine, no difference in tumor multiplicity was seen for either female or male rats lacking VDR, compared to their wild-type counterparts (*Apc^Pirc/+^ VDR^+/+^ n*=36, *Apc^Pirc/+^ VDR^−/−^ n*=36, *P*=0.09). (B) Similarly in the colon, no difference in tumor multiplicity was detected between animals with or without intact VDR expression (*Apc^Pirc/+^ VDR^+/+^ n*=36, *Apc^Pirc/+^ VDR^−/−^ n*=36, *P*=0.2). (C) No increase in tumor size with loss of VDR expression was detected in female rats (*P*=0.7), while male rats showed a significant reduction in tumor size (*Apc^Pirc/+^ VDR^+/+^ n*=67, *Apc^Pirc/+^ VDR^−/−^ n*=55, *P*=0.02). (D) When *Apc^Pirc/+^* rats were treated with the inflammatory agent DSS (gray circles), colonic tumor multiplicity was significantly increased compared to untreated controls, regardless of VDR genotype (black circles, 12 rats/genotype/group, *P*<0.0001). In the *Apc^Pirc/+^* rat, the means of tumor multiplicity data are not normally distributed and require the use of nonparametric statistics; thus, a two-sided Wilcoxon rank sum test was used. Tumor multiplicities in the *Apc^Pirc/+^* rat show significant sex and age differences; therefore, data were blocked based on Lehman's extension to the Wilcoxon rank sum test and jointly tested for the effects of the test conditions. Data shown as mean±s.d.

Overall intestinal tumor incidence in the *Apc* mutant rat was unaffected by VDR loss: *Apc^Pirc/+^VDR^+/+^* (36/36) and *Apc^Pirc/+^VDR^−/−^* (35/36, *P*=0.3). In the small intestine, *Apc^Pirc/+^VDR^+/+^* rats developed an average of 2.6±2.1 (female) or 9.4±5.7 (male) tumors; *Apc^Pirc/+^VDR^−/−^* rats developed 1.7±1.9 (female) or 8.3±6.9 (male) tumors (*P*=0.09, Fig. 2A). Similarly in the colon, *Apc^Pirc/+^VDR^+/+^* rats developed an average of 2.5±2.2 (female) or 5.1±3.2 (male) tumors, while *Apc^Pirc/+^VDR^−/−^* rats developed 1.9±2.0 (female) or 3.9±3.2 (male) tumors (*P*=0.2, Fig. 2B). Overall, there was no effect of VDR loss on tumor multiplicity in the small intestine or colon of *Apc^Pirc/^*^+^ rats.

Colon tumor area was calculated for a subset of animals from each sex/genotype, as described in the Materials and Methods. While tumor size did not differ for females between *Apc^Pirc/+^VDR^+/+^* (7.2±3.1 mm^2^) and *Apc^Pirc/+^VDR^−/−^* (10.1±8.7 mm^2^, *P*=0.7), tumors from *Apc^Pirc/+^VDR^−/−^* male rats (9.5±6.5 mm^2^) were substantially smaller than from their *Apc^Pirc/+^VDR^+/+^* counterparts (15.5±12.7 mm^2^, *P*=0.02, Fig. 2C).

In the inflammatory model of tumorigenesis, DSS did not induce tumor development in any of the *Apc^+/+^VDR^+/+^* or *Apc^+/+^VDR^−/−^* rats examined (six rats/genotype/sex, Fig. 2D). As expected, DSS increased colon tumor multiplicity in *Apc^Pirc/+^VDR^+/+^* rats (females 9.5±4.0, males 49.7±13.8) versus matched untreated controls (females 1.5±1.4, males 4.8±2.1, *P*<0.0001). DSS treatment had a similar effect in *Apc^Pirc/+^VDR^−/−^* rats (females 16.0±4.0, males 19.5±4.9) compared to their untreated controls (females 1.3±1.0, males 2.0±1.9, *P*<0.0001). However, this DSS-enhancing effect was not exacerbated by loss of VDR expression (*P*=0.7).

### Tumor pathology

Fifty 5 µm sections were made from each tumor examined from 180-day-old rats, with every 10th section (50 µm) stained by Hematoxylin and Eosin for evaluation of pathology. Tumors were graded using a 5-point scale of malignancy: (1) adenoma; (3) intramucosal carcinoma; (5) early carcinoma; intermediate grades 2 and 4 were assigned when a lesion exhibited some but not all criteria for the next grade.

Based on this scoring system, the vast majority of tumors were classified as adenomas, showing an average score of 1.4±0.7 for *Apc^Pirc/+^VDR^+/+^* and 1.3±0.5 for *Apc^Pirc/+^VDR^−/−^* (*P*=1). When rats were given DSS, a shift towards increased malignancy was demonstrated for both genotypes when compared to controls. Despite this anticipated shift, no difference in average pathology score was seen between *Apc^Pirc/+^VDR^+/+^* (2.2±1.0) and *Apc^Pirc/+^VDR^−/−^* (2.1±1.5, *P*=0.45) given DSS.

### Biochemical assay results

**Fig. 3.**
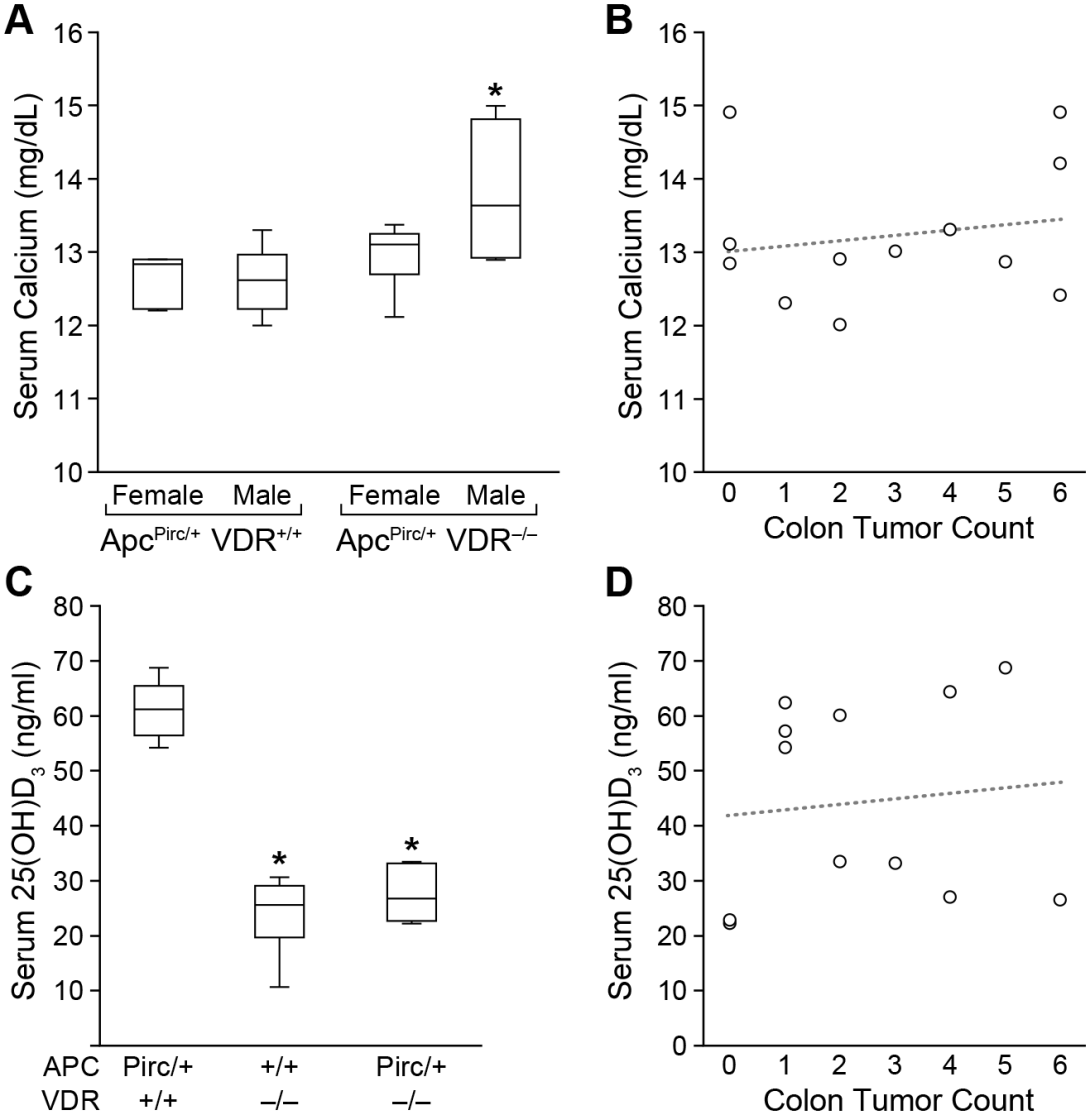
**Biochemical assay data*.*** (A) Serum calcium did not differ between *Apc^Pirc/+^VDR^+/+^* and *Apc^Pirc/+^VDR^−/−^* females (*P*=0.09); serum calcium was significantly increased in *Apc^Pirc/+^VDR^−/−^* males (*P*=0.5, six rats/sex/genotype). (B) No correlation between serum calcium level and colon tumor count was detected (*P*=0.7). (C) Regardless of sex, rats lacking functional VDR showed significantly lower circulating 25(OH)D_3_ level than rats with intact VDR (*P*=0.0001, six rats/genotype). (D) No correlation between circulating 25(OH)D_3_ level and colon tumor count was detected (*P*=1). Serum biochemical assay results were analyzed using a two-sided Wilcoxon rank sum test. * *P*≤0.05. Data shown as mean±s.d.

Serum calcium measured at 120 days of age did not differ between *Apc^Pirc/+^VDR^+/+^* and *Apc^Pirc/+^VDR^−/−^* females (*P*=0.09, Fig. 3A). Males lacking expression of VDR showed elevated serum calcium levels compared to males with intact VDR expression (*P*=0.5). However, no correlation between colonic tumor multiplicity and serum calcium level was found for either genotype (*Apc^Pirc/+^VDR^+/+^ P*=0.5, *Apc^Pirc/+^VDR^−/−^ P*=0.9, Fig. 3B), or when taken as a whole (*P*=0.7).

Circulating 25(OH)D_3_, the commonly measured marker of vitamin D status, was also measured in a subset of samples collected at 120 days of age. Compared to *Apc^Pirc/+^VDR^+/+^* rats (61.1±4.7 ng/ml), rats lacking functional VDR showed significantly lower levels of circulating 25(OH)D_3_ (*Apc^+/+^VDR^−/−^* 23.9±6.5 ng/ml, *Apc^Pirc/+^VDR^−/−^* 27.5±4.4 ng/ml, each *P*=0.0001, Fig. 3C). No evidence for a correlation between circulating 25(OH)D_3_ level and colonic tumor multiplicity was found (*P*=1, Fig. 3D). Of note, even though circulating 25(OH)D_3_ was approximately two-thirds lower in *Apc^Pirc/+^VDR^−/−^*compared to *Apc^Pirc/+^VDR^+/+^* rats, no difference in tumor incidence, multiplicity, size, or pathology was seen.

## DISCUSSION

Our studies to date do not support a role for vitamin D – ligand or receptor – in protection from intestinal tumorigenesis. The current study adds to our body of experimental data, demonstrating no active role for VDR in tumorigenesis in the colon or small intestine. When VDR null rats are placed on a diet that maintains normal serum calcium, the VDR null mutation itself bears no effect on tumorigenesis. In concordance with our findings, Larriba and colleagues found that VDR loss does not impact tumor number in either the small intestine or colon of VDR null *Apc^Min/+^* mice (Larriba et al., 2011). This is also in agreement with our previous finding that vitamin D deficiency does not exacerbate intestinal tumorigenesis when normal serum calcium is maintained (Irving et al., 2018). Furthermore, in the current study, circulating 25(OH)D_3_ is reduced by about two-thirds in VDR-knockout rats compared to wild-type counterparts, yet did not negatively impact tumor outcome.

A role for downstream effects of reduced VDR expression cannot be ruled out. VDR null animals are typically maintained on a ‘rescue diet’ high in calcium and phosphorus, and with the addition of lactose, to maintain serum mineral homeostasis despite the break in vitamin D/calcium signaling. VDR null animals maintained on a typical rodent diet will show significantly reduced serum calcium and increased serum parathyroid hormone, as the calcium homeostatic pathway is interrupted by the lack of VDR feedback. Both Larriba et al. and Zheng et al. found that the loss of VDR expression resulted in larger tumors (Larriba et al., 2011; Zheng et al., 2013). However, in both publications, the diet used to maintain mice contained normal calcium levels and no supplemental lactose, which could plausibly result in decreased serum calcium and/or increased serum PTH in VDR null animals, as demonstrated in our current study and by others (Li et al., 1998). While the use of a non-rescue diet was intentional in the Zheng study, citing evidence that high calcium is known to suppress tumorigenesis, this introduces additional confounders, including impacts to circulating levels of calcium and PTH. In our study, both wild-type and knockout animals received RD to control for this possibility. While RD does suppress circulating 1,25(OH)D_3_, this was true across all groups in our study.

Calcium itself has been implicated in the developmental process for cancer. A meta-analysis of mineral intake and risk for colorectal cancer found that high dietary calcium intake was associated with a significantly decreased risk of colorectal cancer (Meng et al., 2019). Very tightly linked to calcium regulation, PTH has also been linked to cancer development. In the European Prospective Investigation into Cancer and Nutrition (EPIC) cohort, Fedirko et al. found that elevated serum PTH levels were associated with increased risk for colorectal cancer, and that this risk may differ by subsite within the colon (Fedirko et al., 2011). This finding is supported by at least one *in vivo* experimental study, where mice lacking both calcium sensing receptor and parathyroid hormone had increased Wnt/β-catenin signaling and a greater number of aberrant crypt foci after azoxymethane induction than their wild-type counterparts (MacLeod, 2013). Loss of *Apc* function blocks its ability to target β-catenin for degradation, a major contributing factor to colon cancer in humans. This loss of function leads to nuclear β-catenin accumulation and binding to transcription factors that activate *Wnt* target genes. Ligand-bound VDR has been shown to bind to and sequester β-catenin (González-Sancho et al., 2020). Thus, loss of VDR might be expected to exacerbate measures of tumorigenesis in tumors with *Apc* mutation. However, we have shown no deleterious effect on tumor multiplicity, size, nor pathology in animals lacking both proper *Apc* and *VDR* expression, excluding a ligand-independent role for VDR in intestinal tumorigenesis in this model.

Perturbations to normal calcium homeostasis may also help explain why VDR null animals on normal diet have been shown to develop larger tumors in some studies (Larriba et al., 2011; Zheng et al., 2013). Indeed, the only effects on tumorigenesis we have uncovered to date in this line of inquiry correlate with dietary calcium intake or circulating serum calcium levels. We have previously shown that low dietary calcium may be protective against tumorigenesis (Irving et al., 2018). In both the current report in VDR null animals, as well as our previous report with vitamin D deficiency, we do not find evidence for a direct correlation between circulating serum calcium and colonic tumor number. However, here we show preliminary evidence for a reduction in tumor size for the rats with the most elevated serum calcium. By contrast, we have previously shown that hypercalcemia induced by high level vitamin D supplementation showed a positive correlation with increased colonic tumor number; however, in that case we are unable to separate effects of calcium from those of vitamin D (Irving et al., 2015). Deeper investigation into the independent roles of calcium and parathyroid hormone are warranted, especially as disentangled from vitamin D.

Recently, the strength of the causal link between vitamin D and colon cancer has been called into question, and mounting evidence suggests that vitamin D status may simply be association rather than causation. In the *Apc^Pirc/+^* rat, 25(OH)D_3_ serum level significantly decreases over time; however, tumor incidence was 100% at time points prior to the decline, indicating that tumor initiation precedes reduced circulating vitamin D levels. Data in humans supports this notion: He et al. reviewed over 10,000 colorectal cancer cases and 30,000 controls and found no evidence for a causal relationship between 25(OH)D_3_ levels and colorectal cancer risk (He et al., 2018). The authors posit that reported associations are likely the result of unidentified confounders (He et al., 2021).

Vitamin D, mineral homeostasis, and ultraviolet exposure are all inextricably linked in human populations. Even in well-controlled animal studies, these factors can prove difficult to untangle, but it is possible to a much greater degree than is likely to be achieved in humans. Neoplastic phenotype in the *Apc^Pirc/+^* rat closely models multiple facets of the human disease (Ivancic et al., 2013; Irving et al., 2014), and has shown to be both responsive to clinically relevant chemopreventives (Femia et al., 2015), as well as exacerbated by known colorectal cancer-promoting agents (Luceri et al., 2019). Our *in vivo* investigations in this model have found no effect of vitamin D receptor on colon cancer development. Taken together with our earlier publications, as well as recent meta-analyses in humans, previously identified associations between vitamin D and colon cancer risk are likely the result of confounders. Independent effects of calcium, parathyroid hormone, or ultraviolet light exposure remain plausible, and each deserve further exploration.

## MATERIALS AND METHODS

### Development of the VDR knockout rat

The VDR knockout rat (*Rattus norvegicus*) was created by the Genome Editing and Animal Models Core at the University of Wisconsin-Madison. Target sites were selected, and gRNAs were synthesized via *in vitro* transcription, followed by column cleanup and ethanol precipitation purification. One-cell Fischer344.Pirc fertilized embryos were microinjected with a mixture of two gRNA (25 ng/µl each) and Cas9 protein (40 ng/µl; PNA Bio, Newbury Park, CA, USA). Injected embryos were transferred into the oviducts of pseudo-pregnant Sprague Dawley recipients and potential founders born. Weanlings were genotyped and sequenced via subcloning of amplified fragments and Sanger sequencing at the University of Wisconsin-Madison Biotechnology Center. Founders were identified and bred to Fischer344 (F344, Taconic Biosciences) mates to establish an *F*_1_ generation. Heterozygotes from the *F*_1_ generation were then crossed to generate a knockout (KO) population.

### Animal breeding and maintenance

All procedures were approved by the Research Animal Resources Committee of the College of Agricultural and Life Sciences at the University of Wisconsin-Madison. F344 rats were maintained in high density ventilated caging with corncob bedding in the Department of Biochemistry vivarium, with a 12 h:12 h light:dark cycle and free access to food and water. The *Apc^Pirc/+^* rat contains a truncating mutation in the *Apc* gene and is a model of both familial and sporadic intestinal tumorigenesis (Amos-Landgraf et al., 2007; Irving et al., 2014). Multiple intestinal adenomas form as a result of this mutation, with more tumors generally forming in the colon rather than the small intestine, which is similar to the disease pattern in humans. The *Apc^Pirc/+^* line and *VDR^−/−^* line were each maintained through breeding with F344/NHsd rats (Envigo, Dublin, VA, USA); *Apc^Pirc/+^VDR^−/−^* rats were generated by crossing of the two lines for several generations. Genotyping was performed by Transnetyx (Memphis, TN, USA) using real-time PCR.

Breeder and study rats were fed an RD containing 2% calcium, 1.2% phosphorus, and 20% lactose (Envigo, Dublin, VA, USA). This diet is required to aid calcium absorption to maintain normal mineral balance in the absence of functional vitamin D signaling in VDR null rats and was also used in wild-type rats to ensure consistency in diet composition. A subset of animals was used for phenotyping of the newly created VDR null rat; these animals were placed on an ND containing 0.47% calcium, 0.3% phosphorus, and no lactose, with the expectation that these animals would no longer be able to maintain normal serum calcium and PTH levels if the function of VDR were truly disrupted.

### Experimental design

Rats were bred to obtain four genotypes for studies: *Apc^+/+^VDR^+/+^, Apc^Pirc/+^VDR^+/+^, Apc^+/+^VDR^−/−,^* and *Apc^Pirc/+^VDR^−/−^.* Litter-matched animals were used whenever possible to reduce litter- or parent-specific confounders. To induce inflammation-associated colonic tumorigenesis, a separate set of rats was given two rounds (each lasting 1 week) of 4% (wt/vol) dextran sodium sulfate (DSS, FisherSci) separated by 1 week on regular drinking water. Rats were euthanized at 120, 150, or 180 days of age, depending on study needs.

### Tumor phenotyping

At termination, the small intestine and colon were removed, flushed with 70% ethanol, and laid flat. The entire intestine was fixed with 10% neutral buffered formalin for 48 h and then transferred to 70% ethanol until further use. Total tumor counts for the small intestine and colon were obtained by an observer blinded to genotype and treatment groups using a dissecting microscope at 10× magnification. Tumor dimensions were measured to the nearest tenth millimeter using an eyepiece reticule at 10× magnification. Two measurements were taken in the plane of the epithelium for each tumor: the longest tumor dimension was measured first (A), followed by a second measurement (B) perpendicular to the first. To determine an area for each tumor, the equation for area of an ellipse was used: π(A/2)(B/2), as described previously (Irving et al., 2015).

### Tumor pathology

After tumor counts were obtained from fixed sections, samples were collected for histology and reviewed by a pathologist familiar with tumors in the *Apc^Pirc/+^* rat model and blinded to treatment groups. At least nine representative tumors from four to five rats per group were selected for sectioning. Tumors were bisected vertically through the stalk, embedded, and cut into 50 5 µm sections (a total of 250 µm). Every 10th section (50 µm) was stained by Hematoxylin and Eosin for evaluation of pathology. Tumors were graded using a 5-point scale of malignancy: (1) adenoma; (3) intramucosal carcinoma; (5) early carcinoma; intermediate grades 2 and 4 were assigned when a lesion exhibited some but not all criteria for the next grade.

### Biochemical assays

Blood was obtained under ether anesthesia from the ventral tail artery at various time points, and at termination by cardiac puncture to assess circulating calcium, 25(OH)D_3_, and PTH concentrations. Serum calcium was measured by atomic absorption spectrometry on the Perkin Elmer 900H instrument. Serum 25(OH)D_3_ concentrations were measured by the DiaSorin Liaison 25 OH Vitamin D Total Assay (Stillwater, MN, USA). Serum PTH was measured using the Quidel Rat Intact Parathyroid Hormone ELISA (San Diego, CA, USA).

### Protein isolation and detection

Rats were euthanized by CO_2_ asphyxiation. The small intestine and colon were removed and washed with ice-cold sterile PBS plus 1% (vol/vol) HALT protease inhibitor (Thermo Fisher Scientific). Samples were snap-frozen in liquid nitrogen and preserved at −80°C until protein extraction. Samples were thawed on ice and 50 mg added to 1 ml TOTEX lysis buffer, containing 1% (vol/vol) 0.1 M DTT and 1% HALT (O'Connor et al., 2004). Samples were sonicated for 5 s intervals until homogenized, then incubated for 1 h at 4°C. Protein concentration was determined using the Bradford protein assay (Bio-Rad). 30 μg of total protein from tissue lysate was added with equal volume 2xSDS to a 10% polyacrylamide gel electrophoresis system, then transferred to a nitrocellulose membrane (GE Healthcare). The membrane was then washed in PBST, blocked with 5% BSA in PBST, and incubated overnight at room temperature with mouse monoclonal VDR antibody (D-6, sc-13133, Santa Cruz Biotechnology) diluted 1:1000 in PBST. After washing, the membrane was incubated for an additional 2 h in ECL anti-mouse IgG horseradish peroxidase linked whole antibody (GE Healthcare) diluted 1:2500 in PBST. Following successive washes, the membrane was analyzed by enhanced chemiluminescence detection using a Curix 60CP film processor, as described by the manufacturer (GE Healthcare).

### Statistical methods

All statistics were performed using the freely available MSTAT software (Drinkwater, 2022). In the *Apc^Pirc/+^* rat, the means of tumor multiplicity data are not normally distributed and require the use of nonparametric statistics; thus, a two-sided Wilcoxon rank sum test was used. Tumor multiplicities in the *Apc^Pirc/+^* rat show significant sex and age differences (Amos-Landgraf et al., 2007); therefore, data were blocked based on Lehman's extension to the Wilcoxon rank sum test and jointly tested for the effects of the test conditions (Lehmann, 1998). For tumor multiplicity data, 36 rats per genotype were analyzed, giving 90% power at a significance level of 0.05 to detect a change a 50% change in multiplicity in either the small intestine or colon. For animals given DSS, 12 rats per genotype were analyzed, giving 90% power at a significance level of 0.05 to detect a 30% change in colon tumor multiplicity. For tumor sizing, over 50 tumors per genotype were measured, giving 90% power at a significance level of 0.05 to detect a change of 25% in tumor dimension. Data are shown as mean±s.d. Serum biochemical assay results were analyzed using a two-sided Wilcoxon rank sum test. To test for an association between tumor multiplicity and biochemical assay measures, a two-sided Kendall's rank correlation test was used. A *P*-value of ≤0.05 was considered significant for all tests.

## Ethics approval and consent to participate

Animals were housed in a facility inspected and approved by the Association for Assessment and Accreditation of Laboratory Animal Care (AAALAC). All procedures were approved by the Research Animal Resources Committee of the College of Agricultural and Life Sciences at the University of Wisconsin-Madison.
